# Supplementary material for: Effects of NADPH Oxidase Isoform-2 (NOX2) Inhibition on Behavioral Responses and Neuroinflammation in a Mouse Model of Neuropathic Pain
Source: Biomedicines. 2023 Jan 31;11(2):416. doi: 10.3390/biomedicines11020416 (PMC9953009; doi:10.3390/biomedicines11020416)
Supplement: Supplementary file 1 [file biomedicines-11-00416-s001.zip › Figure_S1.pdf]

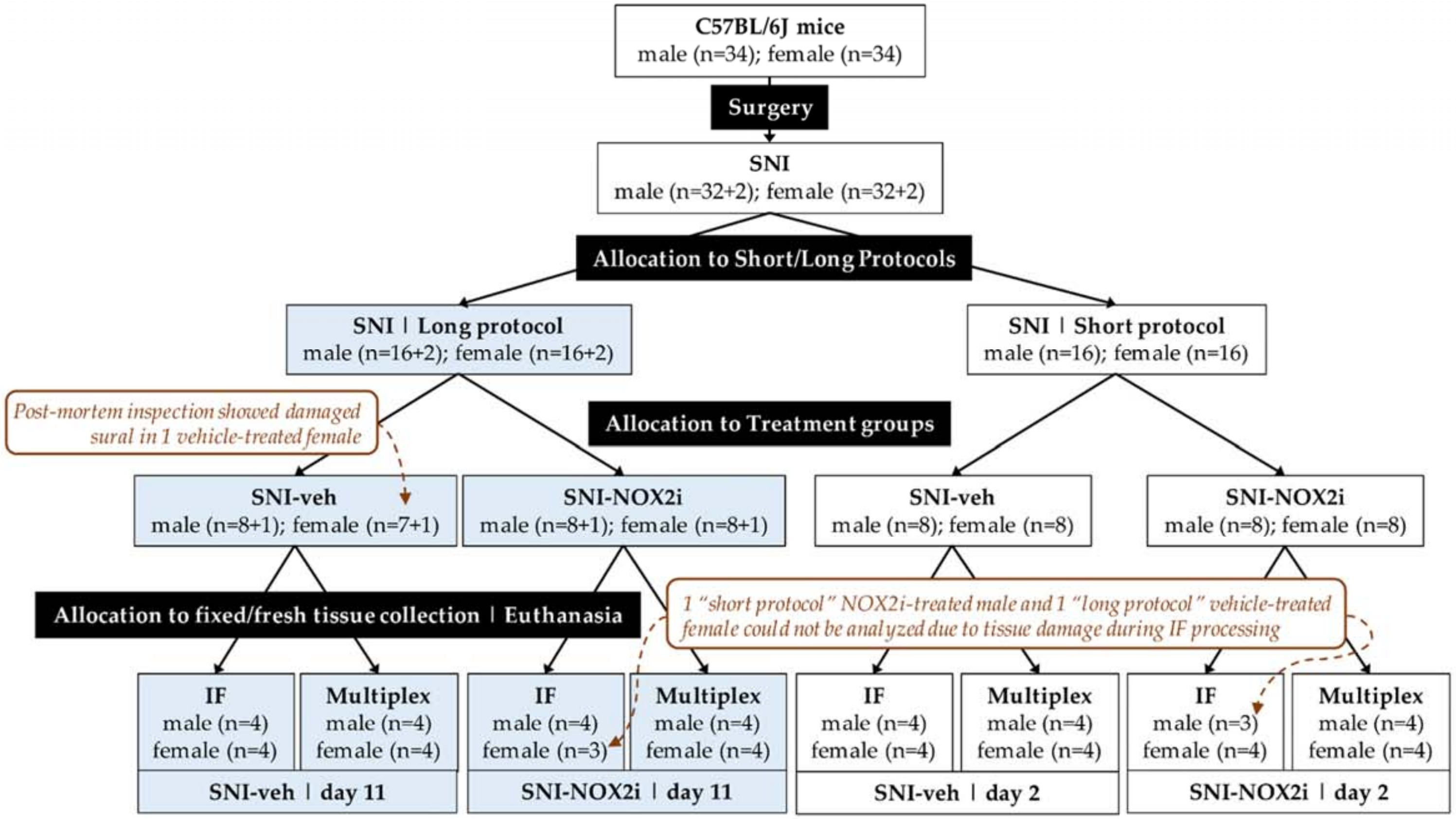

Long protocol animals

Behavioral assessment	SNI-veh		SNI-NOX2i	
	male	*female*	*male*	*female*
Burrowing activity	9	7	9	9
von Frey test	7	6	7	7
LDB test	7	6	7	7

1 vehicle-treated female did not burrow

Animals from 1 of the 8 sets could not be submitted to von Frey and LDB tests due to logistical constraints in the animal facility; the additional set of animals (part of a pilot study for a future work), was only assessed for burrowing behavior

Figure S1. Flow chart showing the number of animals initially allocated to the study, randomly assigned to each experimental group, used in each experimental procedure and included in the analyses, with explanation for exclusion of animals/data points or other missing data. IF, immunofluorescence; LDB, light/dark box; NOX2i, NOX2-selective inhibitor, GSK2795039; SNI, animals submitted to the spared nerve injury surgical procedure. Animals from the "short protocol" were euthanized 2 days after SNI/sham surgery, whereas animals from the "long protocol" were euthanized 11 days after SNI surgery. Only animals from the "long protocol" were subjected to behavioral assessment.
